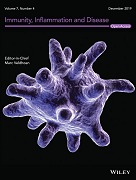# Issue Information

**DOI:** 10.1002/iid3.230

**Published:** 2019-11-10

## Abstract